# “It is a thin line to walk on”: Challenges of staff working at Swedish immigration detention centres

**DOI:** 10.3402/qhw.v10.25196

**Published:** 2015-03-31

**Authors:** Soorej J. Puthoopparambil, Beth M. Ahlberg, Magdalena Bjerneld

**Affiliations:** 1 Department of Women's and Children's Health, Uppsala University, Uppsala, Sweden; 2 Skaraborg Institute for Research and Development, Skövde, Sweden

**Keywords:** Immigration detention staff, emotional dilemma, irregular migrant, fear, administrative detention

## Abstract

Detention of irregular migrants awaiting deportation is widely practiced in many countries and has been shown to have profound negative impact on health and well-being of detainees. Detention staff, an integral part of the detention environment, affect and are affected by detainees’ health and well-being. The objective of the study was to explore experiences of staff working at Swedish immigration detention centres. Fifteen semi-structured interviews were conducted with staff in three Swedish detention centres and were analysed using thematic analysis. The results indicate that the main challenge for the staff was to manage the emotional dilemma entailed in working as migration officers and simultaneously fellow human beings whose task was to implement deportation decisions while being expected to provide humane service to detainees. They tried to manage their dilemma by balancing the two roles, but still found it challenging. Among the staff, there was a high perception of fear of physical threat from detainees that made detention a stressful environment. Limited interaction between the staff and detainees was a reason for this. There is thus a need to support detention staff to improve their interaction with detainees in order to decrease their fear, manage their emotional dilemma, and provide better service to detainees. It is important to address staff challenges in order to ensure better health and well-being for both staff and detainees.

Detention of irregular migrants to enforce deportation is practiced by many governments (Steel, Liddell, Bateman-Steel, & Zwi, 2011). Asylum seekers can also be detained for various administrative reasons such as verifying their claim or identity documents (Mendonça, 2010; Silove, Austin, & Steel, 2007; Steel et al., 2011; UNHCR, 2012). According to international guidelines, immigration detention, also known as administrative detention, should be used as a last resort (EU, 2013; UNHCR, 2012). However, in many countries, migrants are systematically detained in prisons, temporarily constructed structures, at entry points such as airports, or in specialized detention facilities (EU, 2013; Mendonça, 2010; Steel et al., 2011; Venters, Dasch-Goldberg, Rasmussen, & Keller, 2009). The material conditions in detention centres can vary from appalling in some countries to comparatively better standards in others (European Committee for the Prevention of Torture and Inhuman or Degrading Treatment or Punishment [CPT], 2009, 2011).

Studies have shown harmful effects of detention on detainees’ mental (Robjant, Hassan, & Katona, 2009; Steel et al., 2011) and physical health (Venters et al., 2009). In addition to the restriction on liberty, detention environment plays a major role in aggravating or mitigating the effect of detention on health and well-being of detainees (Silove et al., 2007; Silverman & Massa, 2012; Venters et al., 2009). Staff–detainee interaction is a major factor influencing the detention environment (Hall, 2010; Robjant et al., 2009). Staff behaviour such as arbitrary use of power and poor response to queries by detainees can make detainees feel treated less humane. On the other hand, staff might also be exposed to detainees’ frustration and acts of resistance because they are detained and deported against their will. Staff and detainees affect each other's health and well-being in similar ways as in other coerced confined environments such as psychiatric asylums and prisons (Camuccio, Chambers, Valimaki, Farro, & Zanotti, 2012; Finney, Stergiopoulos, Hensel, Bonato, & Dewa, 2013; WHO, 2007). Even though immigration detention is not defined as imprisonment, detainees experience it as imprisonment (Khosravi, 2009; Klein & Williams, 2012). Studies conducted in prisons have indicated that staff–prisoner interaction is what makes life in prison bearable or unbearable and often outweighs the material conditions in determining well-being and quality of life (Crawley, 2004; Dirkzwager & Kruttschnitt, 2012; Johnsen, Granheim, & Helgesen, 2011; Liebling, 2007; Nylander, Lindberg, & Bruhn, 2011; WHO, 2007). As in prisons (Crawley, 2004), staff–detainee interaction can be highly demanding for detention staff due to their constant face-to-face interaction with detainees. Poor levels of interaction with inmates and a highly volatile environment can lead to increased stress and ill health for inmates and staff (WHO, 2007).

Exploring the staff–detainee interaction is vital in understanding how it influences health and well-being of both parties. Experiences of detainees living in Swedish immigration detention centres have been described elsewhere (forthcoming publication).

## Swedish detention centres

The Swedish Migration Board (SMB) has overall responsibility for detention and deportation of irregular migrants and runs all five detention centres in the country with a total capacity of 235 places. In 2013, 2864 individuals were detained of which 1.5% were children and 12% were females (Swedish Migration Board, 2014). The majority of the detainees are rejected asylum seekers whose asylum requests are denied and are required to leave Sweden. Among detainees, there are irregular migrants (migrants without a permit to stay in the country) who have served prison sentences and are then transferred to detention centres for deportation. Detention cases are usually managed by SMB. However, if SMB find detainees not cooperating or deems it necessary to use force to carry out deportation, the case is handed over to police who implement the deportation decision. However, the detainee remains accommodated at SMB's detention facilities. There are no police or security guards inside the detention centres.

Unlike in some other countries, where prison buildings are used for immigration detention (Hall, 2010; Silove et al., 2007; Venters et al., 2009), in Sweden, detainees are accommodated in specialized facilities that have minimal structural resemblance to prisons and detention staff do not wear uniforms. Detainees share sleeping rooms with two to five other detainees and have access to common showers and toilets. They also have access to internet, library, television, and laundry facilities at the centres. Electronically locked doors and high walls in courtyards maintain security in the centres. Staff carry a communication device which can be used as an alarm in case of emergencies (Swedish Migration Board, 2010).

The staff come from different ethnic, educational, and professional backgrounds. The three main categories of staff in regular contact with detainees are supervisors, case officers, and team leaders. They work in teams and shifts. As a minimum requirement for the job, supervisors should have completed secondary education and case officers should have a bachelor's degree in an area such as law or behavioural science. A team consists of two to three supervisors, one to two case officers, and a team leader. Supervisors are responsible for social aspects and housekeeping of detention centres including organizing indoor and outdoor activities, and provision of food, clothes, and other daily necessities. They also coordinate activities with Non-Governmental Organizations (NGOs) who visit centres to provide psychosocial support for detainees. Case officers handle detention cases and are responsible for implementation of deportation decisions. Together with supervisors, they try to motivate detainees to cooperate with deportation while providing humane service. They are responsible for security and managing visits for detainees at the centres. The team leader coordinates activities within and between teams.

## Conceptual framework—emotional labour

In an attempt to explain the experiences described by the staff, we found emotional labour described by Hochschild (1983, p. 7), “the management of feeling to create a publicly observable facial and bodily display,” to be relevant. In other words, emotional labour is the psychological efforts required by an employee to display emotions expected by the work role (Zapf, 2002). Emotional labour is performed mainly in service professions such as nursing (Bakker & Heuven, 2006), police (Bakker & Heuven, 2006; Schaible & Gecas, 2010), and prison officers (Nylander et al., 2011) where there is regular face-to-face interaction with clients (Hochschild, 1983; Wharton, 1999; Zapf, 2002). The difference or inconsistency between displayed emotion and employees’ true inner feeling is called *emotive dissonance*, which if maintained over an extended period can lead to alienation, cynicism, depersonalization, and burnout (Bakker & Heuven, 2006; Hochschild, 1983, p. 90; Nylander et al., 2011; Schaible & Gecas, 2010; Zapf, 2002) resulting in reduced levels of job satisfaction and quality of service delivered. Hochschild (1983) studied emotional labour among flight attendants and bill collectors. Flight attendants have to be always pleasant to passengers irrespective of their behaviour. Similarly, bill collectors are not supposed to show any signs of empathy towards their clients because that may prevent them carrying out their duty; collecting debts.

In order to manage emotional labour, there are two types of acting described by Hochschild (1983); *deep acting* and *surface acting*. Deep acting is where employees alter their inner feelings to conform to organizational norms and the act is thus felt as a representation of inner self. Deep acting is further divided into passive and active acting (Hochschild, 1983; Kruml & Geddes, 2000). Employees spontaneously feel the desired emotion in *passive deep acting*. For example, a prison officer interacting with a young prisoner can feel empathy if the prisoner reminds the officer of his/her own child. Others have referred to this phenomenon as *automatic emotion regulation* (Zapf, 2002) because the emotion was felt spontaneously without any labour (acting). *Active deep acting* is when employees use their past experiences or training to muster a display of the desired emotion. A flight attendant treating an unpleasant passenger as an attention seeking kid is an example.

*Surface acting* is when the act feels “put on” and does not correspond to the true feelings of the employee. This happens when employees act according to the organizational norms and not according to their own norms. Surface acting is associated with emotive dissonance and deep acting is generally associated with better occupational health and job satisfaction (Hochschild, 1983; Nylander et al., 2011; Zapf, 2002). However, one way of acting cannot be considered better than the other (Hochschild, 1983; Schaible & Gecas, 2010). Surface acting may lead to employees suppressing their feelings or alienation and cynicism towards clients but may protect them from being emotionally exhausted. On the other hand, deep engagement resulting from deep acting can lead to work overload, exhaustion, and burn out and might negatively affect the service provided (Schaible & Gecas, 2010).

If properly managed, emotional labour has favourable consequences such as increased job satisfaction, psychological well-being, security, and decreased stress; but if left unchecked, emotional labour may lead to unfavourable consequences such as role alienation, self-alienation, cynicism, and burnout (Kruml & Geddes, 2000). Earlier studies have identified the presence of emotional labour among immigration case officers evaluating residence permit requests (including asylum requests) and the role played by officers’ emotions in performing their jobs (Eggebø, 2013; Wettergren, 2010).

Detention staff is required by the Swedish Migration Board (2010) to provide humane service to detainees while enforcing deportation orders. They have face-to-face interaction with detainees almost every day and are expected to provide professional service to them with empathy, but without being emotionally involved. Seen through the lens of emotional labour, attempts made by staff in accomplishing both aspects of the job is emotional labour and this can give rise to emotive dissonance, if staff considers deportation as a disproportionate measure, forcing them to act contrary to their true inner feeling. The concept of emotional labour can assist in understanding this emotional conflict and efforts by the staff to manage the conflict.

## Objective

The objective of the study was to explore and describe experiences of detention staff in providing services for immigrant detainees. The study is part of a larger project aimed at identifying factors, which could mitigate the effects of detention on the health and well-being of detainees in Swedish immigration detention centres.

## Method

A pilot study on detainee experiences was conducted by the first author as part of his master's education in international health in 2010. Results from this study were discussed with SMB and NGOs, which led to initiation of the current research project.

Permission to conduct interviews at detention centres was sought through emails sent to all the detention centres in Sweden. The first three centres to respond were chosen for data collection. The first author continuously visited the centres and spent 1–2 weeks in each centre during which the interviews were conducted. About 2 weeks prior to the visit, details about the study and an invitation to participate was displayed in the detention centres.

### Study participants

The inclusion criterion for participation was at least 6 months of employment in a Swedish detention centre. The staff present at the centres during the visit were invited to participate. Those who declined the invitation said they would get back later, but never did. Others said they were not interested or lack of time as a reason not to participate. Fifteen semi-structured interviews were conducted with staff members (six females and nine males) including four supervisors, seven case officers, and four team leaders. Duration of interviews ranged from 45 to 75 min. Participants belonged to the age group 25–60 years. Their educational backgrounds included health care, social work, law, political science, developmental studies, economics, and business administration. Their employment period ranged from 6 months to 8 years. Participants’ countries of origin were Angola, Bosnia, Poland, Russia, Sweden, and the former Yugoslavia, but they all spoke Swedish and English.

### Data collection and analysis

Interviews were conducted in English or Swedish in a private room in the centres. The interview guide, which was tested during the pilot study, focused on the participants’ professional background, perceptions about the detention system, interaction with detainees, challenges in carrying out the job, and opinions about on-job training. Data collection was stopped when similar answers to interview questions started to appear during the interviews and no new information was expected to be obtained from conducting further interviews. All the interviews were audio recorded and transcribed verbatim. The transcripts were counterchecked by the first and last authors. Swedish interviews were translated to English by a certified translator. NVivo 10 software was used to manage the dataset (QSR International Pty Ltd, 2012).

**Figure 1:**
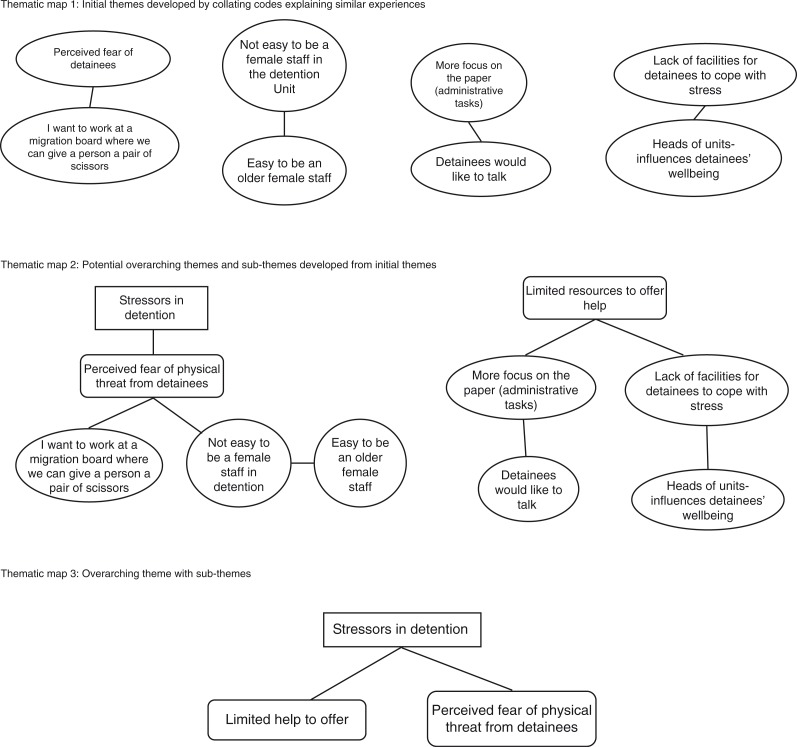
Illustration of the analytical process of developing an overarching theme from initial themes.

Thematic analysis described by Braun and Clarke (2006) guided the data driven analytical process. The first author read all transcripts to develop an overall understanding and further familiarize with the data. Codes identifying patterns in the dataset were developed as exemplified in Table I. Initial themes were formed by collating codes explaining similar experiences. They were then reviewed and organized to develop potential overarching themes and sub-themes. At this stage, all authors discussed the analysis and themes obtained from it and revised the same resulting in three overarching themes and four sub-themes as shown in Table II. Figure 1 shows an example of the analytical process. Throughout the analysis, there was constant reviewing and refining. Codes and sub-themes were reviewed and refined within each theme and later, overarching themes were reviewed and refined in relation to the entire dataset to ensure they reflected the data set.

**Table I:** Coding process.

Interview extract	Coded for
…I think we are better than, for instance [country X]. I read an article from [country X] in Time magazine, and the picture was so worrying.	Swedish detention system better than others

…I think it is because many people who stay here are from Arabic countries. In that culture Mama is a very high person. You don't do your Mama anything.	Respect for older female detention staff

**Table II:** Overarching themes and sub-themes.

Overarching theme	Sub-theme
Immigration detention: not the best management	

Stressors in detention	Perceived fear of physical threat from detainees Limited help to offer

It is a thin line to walk on	The balancing act Clear communication needed

### Ethical consideration

Ethical approval (Dnr 2011/463) was obtained from the Regional Ethical Review Board, Uppsala, Sweden. The participants were informed both in verbal and written formats that their participation was voluntary, only the research team had access to the data collected, and interviews were audio recorded for transcription and analysis. Contact details of the research team were provided to all participants. A written consent was obtained from all participants.

## Results

The results section illustrates the emotional dilemma experienced by the participants and their strategies to manage it while performing their duties. The perceived fear of potential physical threat from detainees influenced their work.

### Immigration detention: not the best management strategy

The staff did not consider detention as an optimal solution but rather as an acceptable strategy for managing irregular migrants for lack of better alternatives. They said detainees were well treated, especially when compared to other countries, and detention was seen as helping detainees to realize there was nothing more they could do to stay in Sweden and thus had to cooperate with deportation. Some of the participants said they, during the initial period of their employment, were not completely in favour of detaining immigrants. But after receiving on-job training and working at detention centres for a while they understood the need for such a system to ensure that everyone followed the law.

I don't really like it because I think it shouldn't be necessary to have places like this. But the system is like this and there are so many people who are not following the decisions. We need to keep them somewhere until they can go home. (Female staff 1)

However, some indicated detention was not the best way to manage irregular migrants, especially when the authorities misuse detention. Misuse, as described by the participants, is detaining an immigrant when it is expected (based on previous experience) that enforcement of the deportation would take time thus prolonging detention duration. This according to them is a waste of resources and is detrimental to detainees.

### Stressors in detention

The perceived fear of potential physical threat from detainees was the main stressor while limited help to offer to detainees made their job challenging.

#### Perceived fear of physical threat from detainees

Though incidents of detainees attacking staff were said to be uncommon, there was still a constant awareness that detainees were not there voluntarily and there is thus always a risk for unpleasant things to happen. This made the detention environment stressful. A majority of the participants expressed fear of being assaulted by detainees, although none of them reported having experienced an assault from detainees. The staff reported being highly dependent on their colleagues for safety because they were the immediate help if something went wrong. Physical security measures were said not to be of much help. They instead focus on “dynamic security” which is based on knowing their surroundings and people therein, thus being able to feel safe and anticipate any event that may occur.

I think that the most important thing for me is dynamic security. I must have a relationship [connection] with all the detainees. It doesn't help that much with the [physical] security and routines. It is important, but there are 25 of them and four of us, if they do something we don't have a chance. That's why it is important to have a good relationship with everyone. (Female staff 4)

The participants, however, reported having little time to acquaint themselves with detainees and cited this as a reason for their fear. Some of the participants, both male and female, considered it challenging for female staff to work in detention. Younger female staff reported being challenged by comments from male detainees on how they dress and conduct themselves. However, there were female participants who said it was an advantage to be a female because detainees were likely to respect and listen to them better. Older female participants reported not facing any problems because they considered most detainees respected them as “Mamma.” Male participants did not discuss their sex as being an advantage or disadvantage for their job.

#### Limited help to offer

The staff expressed concern over lack of facilities for detainees to cope with stress. According to them, detainees found some comfort by talking to staff and fellow detainees. Detainees mostly spoke about their legal cases, families, and the unfortunate situation they were in.

Talking and spending time with detainees helped the staff to proactively identify and assist detainees and to let them know staff listened and cared for them. However, most of the participants reported not having enough time to spend with detainees due to high amount of administrative tasks except during nights when they had fewer tasks. A major concern for the staff was absence of a counsellor at the detention centres with whom detainees could freely interact. They expressed the need to have more training in communication strategies, refresher courses, and workshops with staff from other detention centres to improve services provided.

The participants appreciated visits by volunteers from NGOs because talking to a person not working for SMB often seems to ease detainees’ stress. They indicated the wish to have more cooperation with volunteers in order to better understand and help detainees. Some participants were, however, cautious about volunteers giving false hopes to detainees because they could do little to influence the outcome of detention cases.

Lack of social and educational activities which could help detainees cope with stress was another concern expressed by the participants and was partially attributed to restrictions such as limited access to gym or courtyard at the centres. Safety regulations prevented detainees from cooking, gardening, or carpentry. Some participants considered security measures at the detention centres to be too restrictive which made them and detainees more stressed.

According to the participants, heads of detention centres could relax some of the restrictions. For example, facilities allowed in one centre which were said to positively influence the well-being of detainees were prohibited in another based on the management's decision.

It's the local management … in [detention unit X] … the women there can colour each other's hair. They can bring in hair products and they think it's a good way for them to feel better to have the ‘beauty time’ … It is something to do for them and makes them feel good … And here it is strict regulations they cannot bring in hair colour at all because they say it is dangerous. (Female staff 6)

### It is a thin line to walk on

This theme describes the challenges experienced by the staff in being a migration officer and simultaneously a fellow human being and strategies used to overcome these challenges.

#### The balancing act

The participants said it was important to be both formal (migration officer) and informal (fellow human being) with detainees. However, they all found it challenging to balance both roles.

The first thing you have to bear in mind when you work here is that you are a civil servant for the Swedish migration authority. You are on duty … But on the other hand there are people who are locked in here, in custody. You have to be a human … So you are just to set a line between your professional and your social emotions. It is sometimes very complicated and difficult. But we try to … balance so we don't fall into emotional things. (Male staff 1)

The formal part of the balancing act was reportedly intricate when the participants doubt decisions taken by authorities to detain and deport a migrant. This creates emotional conflicts for them because they might be implementing a disproportionately taken decision. Meanwhile, the participants tried to motivate detainees to cooperate with deportation by encouraging them to be hopeful about life after deportation, even though they were not always sure about the consequences of deportation and thus performing an untrue act. Being a fellow human being similarly created emotionally difficult situations, especially when it came to detention of vulnerable individuals such as elderly, sick, and children. Deporting a detainee whom the participants believed to have a life-threatening situation in his/her home country proved emotionally challenging for the participants. Playing the informal role created situations such as a detainee requesting help from the staff to escape from detention because the staff was perceived to be friendly.

The staff tried to overcome these challenges by not thinking much about the bigger picture; the detainees’ story or their asylum process. This was reported to prevent them from having emotional conflicts within oneself. If conflicts occur, participants said they consoled themselves by blaming SMB for the decision. Another strategy mentioned in the balancing act was to set boundaries; for example, by not disclosing details of personal life (marital status, children) to prevent detainees getting too friendly.

Irrespective of the participant's personal opinion, believing that SMB has taken the right decision to deport a detainee also helped in balancing the act. According to the participants, this kind of belief is necessary to work in a place like detention, but they also expressed the wish to have the balancing act discussed during their on-job training because it was emotionally challenging.

#### Clear communication needed

The participants stressed the importance of good communication among staff and between staff and detainees in order to perform their duties properly. This is highly important considering that decisions from SMB are written in Swedish which many detainees cannot read or write. The participants said detainees can accept reasons why they were detained and can take “No” for an answer, if clearly communicated with proper reasoning.

Responding to queries from detainees was considered important although the participants also noted they were not always able to do it. A commonly asked question was about duration of their stay in detention and the staff often had no specific answer, especially when deportation order was processed by the police.

The participants also wanted to have more record keeping and clear communication between themselves. According to them, this would mitigate lots of frustration among them and detainees.

… he [detainee] was trying to ask for help yesterday and he asked somebody [staff] but that staff went home. And then he asked somebody [else] during the night, and that staff said “I don't know, maybe.” Then he came to me in the morning and said “I have been asking two three people. Everybody was saying maybe later” … what lots of people [staff] do is that they are listening to the detainee and then they go away and forget it. (Female Staff 2)

## Discussion

The detention staff experienced emotional dilemma in being migration officers and simultaneously fellow human beings while providing service to detainees who are in a difficult situation. They tried to balance their roles. This balancing act is emotional labour because the staff are trying to manage their emotional conflicts in performing their duties. This is why we chose the concept of emotional labour to discuss the staff experience of managing their emotional conflicts to perform their tasks in accordance with organizational norms.

Because studies focusing on detention staff are rare, studies conducted among prison officers are referred to in discussing the study results. There exist differences between prisons and detention centres. Prisons are characterized by tall walls, barbed wires, being locked up in cells, prison officers wearing uniforms, and providing services to criminals or suspected criminals who are usually aware of the duration of their prison sentence. On the other hand, detention centres in Sweden lack structural similarity to prisons (no barbed wires, no cells), detention staff does not wear uniforms and they provide service to immigrants who are not charged with criminal offences but are detained to be deported without knowing the duration of their detention. However, prison officers and detention staff share a common trait; they provide service to people who are confined against their will (Liebling, Price, & Elliot, 1999; Nylander et al., 2011) and the aim of the study was to explore the experiences of detention staff in providing service to detainees.

Being a fellow human being, which is important to provide humane service, can risk the detention staff being deeply involved and emotionally affected by the plight of detainees which could lead to exhaustion and burnout. The detention staff performed emotional labour, the balancing act, to avoid this. They performed an act of self-monitoring (Wharton, 1999) by setting boundaries to control their emotional involvement and prevent exhaustion. Similar strategies are used by nurses to avoid emotional involvement by considering their patients as impersonal objects (Bakker & Heuven, 2006) and by prison officers considering prisoners as merely “bodies” (Crawley, 2004). This prevents their emotional attachment with the client and thus averts burnout. However, if excessively used, this could lead to alienation (Nylander et al., 2011). The staff performed untrue acts (surface acting) when they gave unfounded hopes or provided services to detainees who were disproportionately detained (misuse of detention). They tried to overcome this emotional dissonance either by blaming or believing the righteous system. This is common among prison officers where they tend to attribute their actions to regulations provided by authorities (Bruhn, Nylander, & Lindberg, 2010; Liebling et al., 1999). Wettergren (2010) and Eggebø (2013) discuss emotional dilemma experienced by immigration case officers in Sweden and Norway respectively. Although these officers had only minimal or no face-to-face contact with migrants, they often found themselves in emotionally difficult situations where they would have liked to grant permit to an applicant but regulations suggested the opposite. In such instances, they rejected the application and consoled themselves by reaffirming their faith that the decision was based on the law and not on their moral or emotional judgements. Although our participants tried to manage their emotional dilemmas by performing the balancing act, they often found it challenging.

Work environment influences employees, clients, and their interactions (Hochschild, 1983; Molleman & Leeuw, 2012; Wharton, 1999). It is argued in the literature that improved staff–inmate interaction promotes a calmer detention environment, better understanding, and greater respect between the parties (Dirkzwager & Kruttschnitt, 2012; Leggett & Hirons, 2006; Nylander et al., 2011). However, this demands that staff spend more time with inmates (Bruhn et al., 2010; Crewe, 2011; Malloch & Stanley, 2005). But, most of the participants’ time was spent on administrative tasks. This might be counterproductive because the participants seem to consider dynamic security, focused on knowing ones surrounding and developing good relationships (Leggett & Hirons, 2006), more useful than physical security measures such as electronic locks and alarms (Malloch & Stanley, 2005; Silove et al., 2007). Staff working in confined facilities such as prisons and detention centres found it necessary to interact and listen to their inmates not only for helping and understanding inmates better, but also for their own safety (Duxbury & Whittington, 2005; Hall, 2010; Johnsen et al., 2011; Silove et al., 2007). This is important because lack of safety leads to fear which causes job dissatisfaction and stress (Camuccio et al., 2012; Crawley, 2004; Cullen, Link, Wolfe, & Frank, 1985; Hall, 2010; Liebling, 2007).

The lack of time spend with detainees creates a vicious circle where lack of knowledge about detainees can force staff to focus more on physical security measures instead of dynamic security, which could lead to cynicism and alienation (Liebling, 2007; Nylander et al., 2011). This contributes to the perceived fear of being assaulted, forcing the staff to focus on physical security and not spending much time with the detainees, thus completing the circle. Such distanced interaction can lead to surface acting, emotive dissonance, and fear among staff while leading to suboptimal service for detainees. A study conducted among Swedish prison officers, using the concept of emotional labour, found that fear was more prevalent among staff working in high security prisons than in low security prisons, indicating the limited effectiveness of physical security. In lower and medium security prisons, the prison officers found it easier to explain and make inmates follow the rules (Nylander et al., 2011).

Often, perceived fear among staff has less to do with real danger, but rather with the potential for violence (Cullen et al., 1985). In her ethnographic study conducted in a detention centre in the UK, Hall (2010) discusses this *sporadic reality of violence* and suggests that the root cause of perceived fear among officers in her study was the lack of knowledge about detainees. They were afraid of the constant potential for violence among frustrated detainees who might have had criminal convictions. Similar to Sweden, the UK also detains irregular migrants who have served prison sentences and are waiting to be deported. This puts the staff, in hyper vigilance mode alienating them from detainees. Although not reported by the participants in our study, the presence of detainees with criminal convictions might also be an underlying cause for their perceived fear of physical threat from detainees. The current study does not provide any insight into this and further research is therefore needed before drawing conclusions.

According to the instructions provided by the Swedish Migration Board (2010) and as reported in the results, communication with detainees is one of the most important aspects of detention staff's job description. Better communication between staff and inmates contributes to staff's understanding of inmates and increased dynamic security, which leads to decreased fear (Bruhn et al., 2010; Camuccio et al., 2012; Duxbury & Whittington, 2005). As in the case of prison officers (Crawley, 2004; Nylander et al., 2011), regular interaction with detainees can help the detention staff to improve their relationship with the detainees. In addition to regular interaction with confined individuals, explanation of rules and decisions demand good communication skills, which could be achieved through training (Bruhn et al., 2010; Molleman & Leeuw, 2012; Nylander et al., 2011). This was a repeated request from the participants in this study. Emotional dilemma can come into play during these interactions and if properly adapted, emotional labour could assist them in not becoming “too friendly” (Leggett & Hirons, 2006; Liebling et al., 1999) or “too formal.” Building good relationships and having good interaction is the core feature of dynamic security (Leggett & Hirons, 2006).

Emotional dilemma experienced by immigration detention staff is an inherent part of their job. Immigration case officers in studies conducted by Eggebø (2013) and Wettergren (2010) found it easier to manage their dilemma by discussing it with their peers facing the same challenge. Graham (2002, p. 216) in his study on Swedish civil servants providing assistance to immigrants also found sharing emotional conflicts with peers and reaching emotional consensus helpful in managing emotional conflicts.

Similar to prisons (Crawley, 2004; Dirkzwager & Kruttschnitt, 2012), it is through the detention staff that the idea of “detention” and its severity or softness is being created (Hall, 2010). It is thus important for the organization to discuss the emotional dilemma and balancing act by the staff in order to provide support.

## Conclusion

Detention staff in Sweden is employed to play the role of ensuring that decisions to deport irregular migrants staying in Sweden are implemented. At the same time, as human beings dealing with other marginalized human beings, they are torn between two loyalties: their official work and empathy. This creates emotional dilemma increasing their stress and influences how they interact with detainees. Emotional dilemma becomes an inherent part of their job and they try to manage it through balancing their acts. The staff's perceived fear of physical threat from detainees seems to arise from the nature of their work and the confined work environment. It might also be influenced by the presence of detainees who have criminal backgrounds and have served prison sentences. Detention staff, along with detainees, are part of a contested system and their well-being is highly interdependent. Authorities should address the staff challenges and provide support to the staff. Addressing the emotional dilemma and perceived fear present among staff enables them to improve their service to detainees and ideally, mitigate the effect of detention on the health and well-being of detainees.
